# Integrated clinical research ensembles: A pathway to increased academic productivity

**DOI:** 10.1017/cts.2025.10130

**Published:** 2025-09-02

**Authors:** Sergey Tarima, John R. Meurer, David Friedland, Ndidiamaka Ojiako, Michael Anello, David Zimmerman, Renee McCoy, Reza Shaker

**Affiliations:** Clinical and Translational Science Institute of Southeast Wisconsin, Medical College of Wisconsin, Milwaukee, WI, USA

**Keywords:** Integrated Clinical Research Ensembles, team science, academic productivity, publication metrics, interdisciplinary collaboration

## Abstract

**Introduction:**

The study objective was to evaluate whether the formation and funding of team science-guided Integrated Clinical Research Ensembles (ICREs) enhance individual faculty productivity, measured by publication and impact factor adjusted citation rates. The setting was a multi-institutional NIH Clinical and Translational Science Award-supported hub.

**Methods:**

Monthly faculty publication and impact factor adjusted citation rates were analyzed using data extracted from the hub-managed Faculty Collaboration Database (FCD). The FCD imports indexed publications for all faculty members across four academic institutions, drawing from PubMed and faculty curriculum vitae. Monthly publication counts were modeled using Poisson regression, fitted using generalized estimating equations to account for clustering of observed monthly publication rates of individual faculty. Publication rates were compared before and after ICRE formation and funding, and between faculty in and outside ICREs.

**Results:**

Before joining ensemble teams, ICRE faculty had an 87% higher monthly publication rate than non-ICRE faculty. As ICREs were funded, the monthly publication rate increased an average 72% compared to baseline levels and future citation rates determined by journal impact factors increased by 150%.

**Conclusions:**

Faculty publication and citation rates significantly increased following ICRE funding, demonstrating the potential of structured team science models to boost academic productivity and influence. Faculty inclined to participate in team science through formalized ICREs were already among the more productive faculty.

## Introduction

Addressing unmet health needs of patients and communities requires innovative approaches that transcend traditional research silos. In 2019, Reza Shaker, MD, conceptualized the Integrated Clinical Research Ensemble (ICRE) in the Clinical and Translational Science Institute of Southeast Wisconsin (CTSI), to foster a team science approach focused on solving real-world health challenges. The ICRE model assembles interdisciplinary teams of patients, clinicians, and basic and clinical scientists to collaboratively develop solutions. With rare exceptions, methodologists (such as epidemiologists, biostatisticians, biomedical informaticians, and bioinformaticians) also partake in these teams. These solutions span new medical devices, medications, processes, procedures, and research proposals. The ICREs benefit from project management, resource access, and an initial line of credit. Importantly, the active involvement of patients and clinicians ensures that these solutions are both practical and clinically relevant. Interdisciplinary collaboration often encounters challenges such as disciplinary silos, conflicting incentives, and limited institutional support, which hinder the realization of team science’s full potential [1]. The ICRE model provides a structured framework to address these gaps by fostering cross-disciplinary partnerships, aligning incentives, and providing centralized resources to enhance academic productivity.

The concept of integrated research teams has been supported by evidence linking team-based approaches to improved outcomes in both research productivity and impact. Studies have shown that interdisciplinary collaboration can lead to higher publication rates, more diverse funding portfolios, and stronger scientific innovations [1]. Furthermore, such frameworks improve the translation of research findings into practice by integrating the perspectives of community stakeholders, clinicians, and scientists early in the process [2]. The ICREs represent a practical application of this model, fostering a culture of collaboration that prioritizes patient-centered and community-informed solutions.

Interventions designed to improve research productivity at the group level often focus on structured collaboration, shared resources, and mentorship. Programs like Team Science and Collaborative Research Networks have demonstrated success in boosting research outputs, including publications and grant funding, through formalized support structures and team-based accountability [3,4]. Metrics such as the number of peer-reviewed publications, citation impact, and network strength are commonly used to evaluate the success of such interventions [5]. The analysis of journal impact factors is crucial for evaluating academic productivity and career advancement, as publications in high-impact journals often influence grant funding, faculty promotions, and institutional reputation [6]. By embedding group-level interventions within an institutional framework like CTSI, ICREs have the potential to magnify these outcomes, creating a robust pipeline for academic productivity and career advancement.

Fostering research productivity is a growing challenge in the academic context, particularly in an era emphasizing collaboration and interdisciplinary engagement. Traditional research models, which often isolate investigators, have been criticized for limiting opportunities for high-impact outcomes [7]. The ICRE model addresses this issue by formalizing multidisciplinary teams to enhance academic productivity, with goals including increased publications, grant submissions, and innovative project presentations [3,8] Guided by the CTSI principle of “All in Together,” the ICRE framework aligns with the Mutually Learning Trilateral Ecosystem—a model integrating the healthcare system, the research enterprise, and community stakeholders. As “translational engines” within this ecosystem, ICREs accelerate the development of solutions to pressing patient problems.

From 2019 to 2023, the CTSI ICRE program established 44 pre-ensembles and funded 22 ensembles, engaging 414 team members from CTSI partner institutions and community organizations. The participation of academic faculty—bringing expertise in clinical, basic, and translational research—has been critical to these ensembles’ success.

This manuscript evaluates the impact of ICRE participation on faculty academic productivity, using publication rates as a key metric of career advancement and research success. The study examines the role of ICREs as a transformative model for research productivity, evaluating their impact on publication rates as an indicator of academic success. By analyzing the outputs and experiences of faculty participants, we aim to contribute to the growing body of evidence supporting structured, interdisciplinary research models. This study aims to evaluate the ICRE model’s effectiveness in enhancing publication rates while also examining its impact on citation metrics, providing a comprehensive view of both productivity and scholarly influence.

## Methods

To quantify academic productivity, a metric was needed that was measurable over time, objective, easily accessible, interpretable, and accurate. Additionally, the presence of a corresponding control group or the ability to evaluate the metric in the study population before and after joining an ICRE was necessary. Considerations for metrics included grant submissions or funding, presentations, abstracts, meetings attended, and publications. A reasonable metric satisfying the above conditions was a monthly publication rate, given a large enough number, and our CTSI’s development of the Faculty Collaboration Database (FCD); Wes Rood is the developer of FCD. The FCD was started with an initial upload of faculty curriculum vitae (CVs) and it maintains the CVs by automatically importing publications from online medical bibliographic databases (e.g., MEDLINE and PubMed). An automated process allows faculty to confirm imported publications; only confirmed publications were used in this data analysis. This database allowed for the quantification of monthly publication rates for faculty in, and never in, an ICRE, as well as for publications almost 10 years prior to the development of ICREs.

Many publications were co-authored by multiple faculty members, so we focused on author-publication pairs as the unit of the data analysis. To analyze faculty publication rates, we used these author-publication pairs and calculated monthly publication rates for each faculty. This produced dependent monthly counts within each faculty. These rates were analyzed using Poisson regression with overdispersion, accounting for within-author dependence using generalized estimating equations (GEEs) proposed by Liang and Zeger [9]. This Poisson model also accounted for fixed temporal (publication year) and seasonal (publication month) effects. Using the Poisson model, we compared how ICRE faculty publication rates differed from other faculty prior to joining an ICRE team, after the team was formed (pre-ensemble), and after the ICRE was formally funded.

To incorporate the cited journal’s impact factor, we calculated another measure of academic performance: “Monthly increase in the number of future citations” (MINFS). Since impact factor is calculated as an average number of citations per publication, the new composite endpoint was defined as the sum of impact factors for monthly publications. For example, if a faculty member has 3 publications in January 2019 in journals with impact factors 0.8, 1.1 and 4.9, then their monthly count would be equal to 3, while the composite endpoint MINFS would be equal to 7.8 (=1.8 + 1.1 + 4.9) indicating that these 3 publications will likely generate nearly 8 citations. This measure weighs each publication by the impact factor of the journal where it was published. Then, the monthly publication rate becomes an impact-factor-adjusted monthly publication rate predictive of the number of future citations or the monthly increase in future citations. By analogy with using Poisson GEE for modelling monthly publication counts, monthly increase in future citations was modeled with quasi-likelihood which also used log link function for the mean and variance equal to the mean [9]. A sandwich variance estimator was used to obtain consistent standard errors.

Two distinct populations were evaluated in this study. The largest group, the main comparator group, were those never in an ICRE. This group’s publication rate was examined between 2010 and 2022 and provided a baseline publication rate with adjustment for yearly and seasonal variation. The other population included those ultimately participating in a funded ICRE. This population was evaluated across three specific time frames: (1) prior to forming an ensemble; (2) a pre-ensemble team collaborating to generate preliminary data and prepare an application for ICRE funding; and (3) a team awarded ICRE funding and administrative support. Over the course of the ICRE program to date, 44 pre-ensembles were formed and 22 of them were awarded funding.

## Results

**Figure 1.**
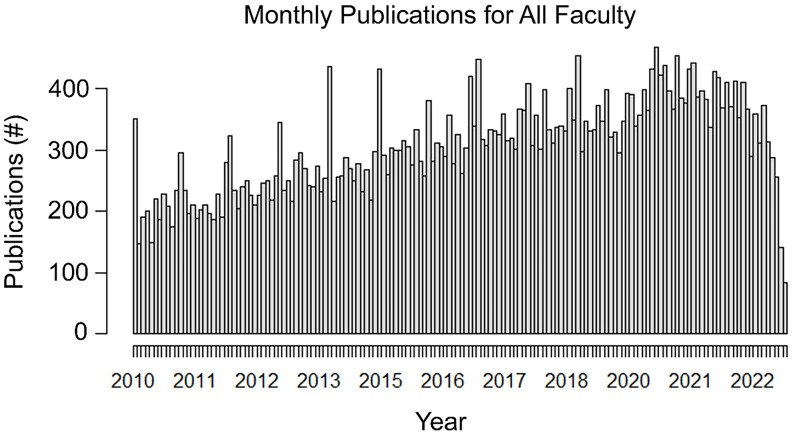
Number of monthly publications for all faculty by year.

There were 414 ICRE members across CTSI who participated in forming 44 pre-ensembles including 22 funded ICREs (Table 1). Non-faculty staff, students, and community members (*n* = 205) and those who did not have publications since 2010 (*n* = 22) were excluded from consideration, leaving 187 faculty ICRE members for analysis. Of 3,279 non-ICRE faculty, 1,512 did not have validated publications in the FCD, leaving 1,767 non-ICRE faculty for comparison. Reasons why some publications are unconfirmed are likely multifactorial and include mismatched and erroneous entries, the inclusion of purely clinical faculty, and some faculty not using the FCD.. Publication frequency increased year over year from approximately 200 monthly in 2010 to nearly 400 monthly in 2022 across the whole cohort (Figure 1).

**Table 1.** Number of faculty and/or ICRE members from each CTSI institution

CTSA Hub Primary Institution	Member of ICRE	Never Member of ICRE
Medical College of Wisconsin	196	3078
University of Wisconsin – Milwaukee	5	93
Marquette University	6	82
Milwaukee School of Engineering (MSOE)	1	13
Versiti Blood Center of Wisconsin	0	5
Zablocki VA Medical Center	0	5
Froedtert Hospital (adult hospital partner)	0	3
Children’s Wisconsin (pediatric hospital partner)	1	0
No entry – Not Faculty	205	0
**Total**	**414**	**3279**

**Figure 2.**
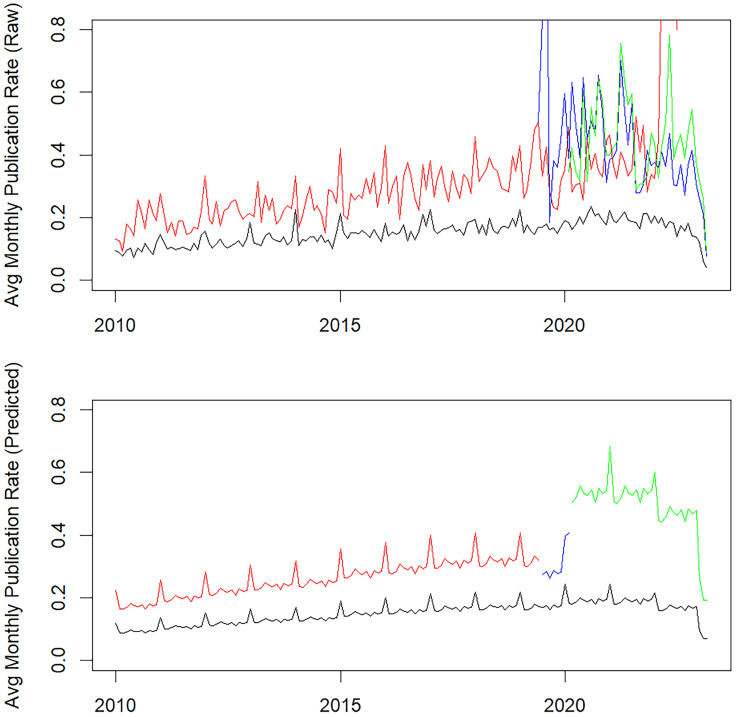
Average Monthly Raw (upper panel) and Predicted (lower panel) Publication Rates by Year (Black are non-ICRE faculty months, Red are ICRE faculty months before joining, Blue are pre-ICRE faculty months, Green are funded ICRE faculty months). The predicted values are plotted for a hypothetical scenario when a team is formed in August 2019 and funded 8 months later.

A GEE-fitted Poisson model controlling for fixed year and month effects was applied (Table 2 and Figure 2). The Poisson regression analysis was based on 265,689 non-ICRE faculty months (black periods), 24,850 ICRE faculty months prior to joining (red periods), 2,035 pre-ICRE faculty months (blue periods), and 2,371 funded ICRE faculty months (green periods). ICRE faculty, even before joining an ICRE, already demonstrated an 87% higher publication rate than non-ICRE faculty with an Incidence Rate Ratio (IRR) = 1.87, *p* < 0.001. The publication rate of ICRE faculty did not significantly change when a pre-ensemble was formed but not yet funded (IRR = 0.87, *p* = 0.537). If the funding was awarded, faculty monthly publication rates received an additional boost with a 72% increase in publication rate (IRR = 1.72, *p* = 0.017). Since this GEE analysis (Table 2 and Figure 2) did not control for clustering associated with ensemble teams, a sensitivity analysis adjusted for ensemble team clustering was performed for a subset of ICRE faculty only, where the same predictor structure was used except for exclusion of the ICRE faculty indicator. The findings were very similar: the publication rate did not significantly change when a pre-ensemble was formed (IRR = 0.9, *p* = 0.675), and awarded funding led to a 77% increase in publication rate (IRR = 1.77, *p* = 0.017).

**Table 2.** Incidence rate ratio (GEE-fitted Poisson regression modelling monthly publication counts)

Variable	IRR	Lower bound of the 95% CI	Upper bound of the 95% CI	*p*-value
YEAR = 2011	1.14	1.08	1.22	<0.001
YEAR = 2012	1.26	1.18	1.35	<0.001
YEAR = 2013	1.37	1.27	1.47	<0.001
YEAR = 2014	1.43	1.33	1.54	<0.001
YEAR = 2015	1.60	1.48	1.73	<0.001
YEAR = 2016	1.68	1.56	1.82	<0.001
YEAR = 2017	1.79	1.64	1.95	<0.001
YEAR = 2018	1.83	1.68	1.99	<0.001
YEAR = 2019	1.83	1.67	2.00	<0.001
YEAR = 2020	2.05	1.87	2.25	<0.001
YEAR = 2021	2.04	1.86	2.24	<0.001
YEAR = 2022	1.80	1.63	1.99	<0.001
YEAR = 2023	0.78	0.67	0.89	<0.001
MONTH = FEB	0.74	0.70	0.79	<0.001
MONTH = MAR	0.74	0.69	0.78	<0.001
MONTH = APR	0.76	0.72	0.81	<0.001
MONTH = MAY	0.82	0.77	0.87	<0.001
MONTH = JUN	0.78	0.74	0.84	<0.001
MONTH = JUL	0.77	0.72	0.83	<0.001
MONTH = AUG	0.80	0.75	0.85	<0.001
MONTH = SEP	0.74	0.69	0.79	<0.001
MONTH = OCT	0.81	0.76	0.86	<0.001
MONTH = NOV	0.78	0.74	0.82	<0.001
MONTH = DEC	0.80	0.75	0.84	<0.001
ICRE FACULTY Prior to forming ICRE	1.87	1.49	2.35	<0.001
ICRE FACULTY Pre-Funding*	0.87	0.56	1.35	0.537
ICRE FACULTY Funded*	1.72	1.10	2.69	0.017

* Compared to ICRE Faculty prior to forming an ICRE. All else is compared to monthly publication rates in January 2010 of faculty never in an ICRE; IRR = Incidence Rate Ratio; the reference category is a non-ICRE faculty in January 2010.

**Figure 3.**
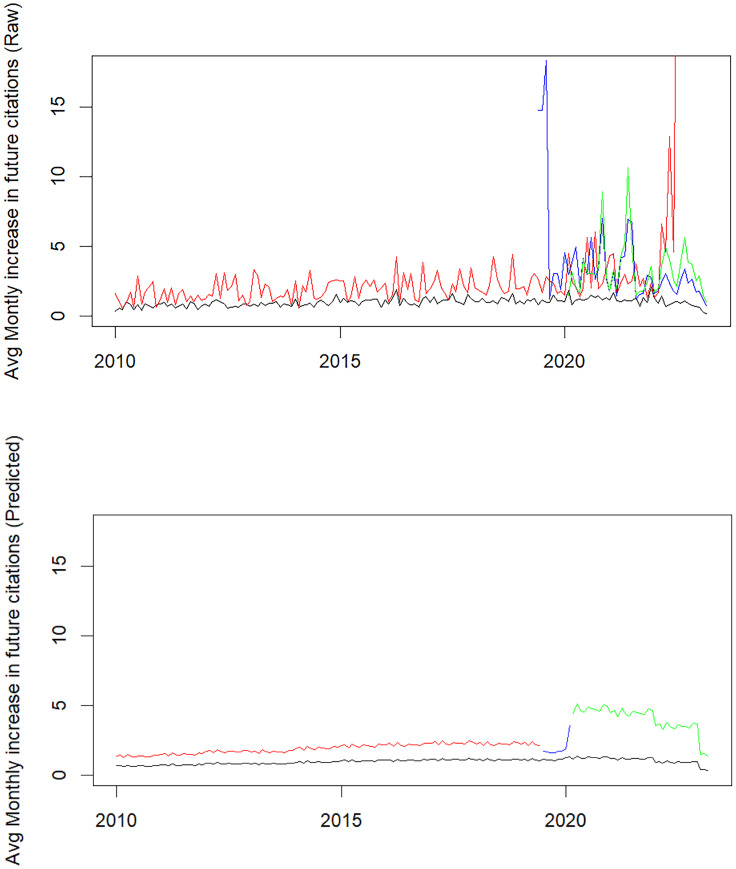
Observed (upper panel) and Predicted (lower panel) Monthly Increases in Future Citations by Year (Black are non-ICRE faculty months, Red are ICRE faculty months before joining, Blue are pre-ICRE faculty months, Green are funded ICRE faculty months). The predicted values are plotted for a hypothetical scenario when a team is formed on in August 2019 and funded 8 months later.

**Figure 4.**
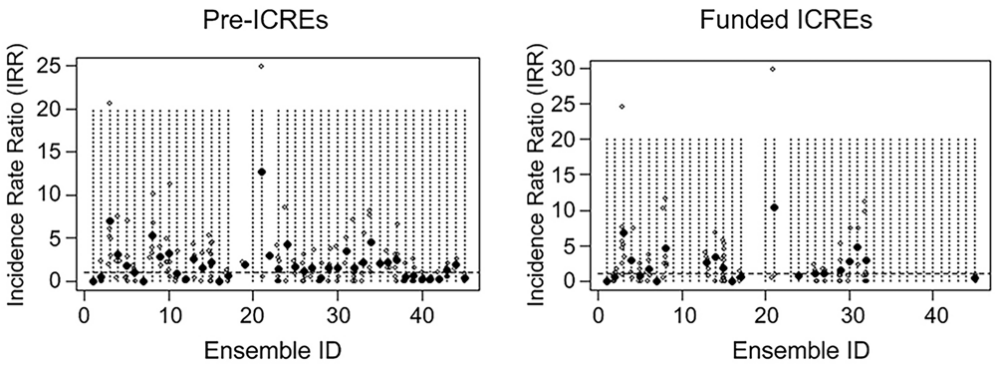
Incidence rate ratios of monthly number of publications for Pre-ICREs and funded ICREs relative to non-ICRE group.

Table 3 summarizes the quasi-likelihood of monthly increase in the number of future citations, which did not significantly change when a pre-ensemble was formed; the multiplicative effect is 0.74, *p* = 0.430. The awarded funding led to a 150% increase of the monthly increase in the number of future citations; the multiplicative effect is 2.5, *p* = 0.013. Figure 3 reports raw and predicted monthly increases in numbers of future citations. Note that 19.1% of all publications had a missing journal impact factor. These publications were excluded from the analysis of monthly increase in the number of future citations. Analysis was performed for individual ICRE teams, both at the initial formation (pre-ensemble) and after funding (Figure 4). An annual IRR at the individual faculty level for publications was calculated and charted. This measure provides insight into the variability between ICREs and among faculty within an ICRE, as to their relative publication productivity.

**Table 3.** Main study. Quasi likelihood model of monthly increase in number of future citations

Variable	RMINFC	Lower bound of the 95% CI	Upper bound of the 95% CI	*p*-value
YEAR = 2011	1.10	0.96	1.28	0.182
YEAR = 2012	1.25	1.09	1.42	0.001
YEAR = 2013	1.24	1.07	1.42	0.003
YEAR = 2014	1.42	1.23	1.63	<0.001
YEAR = 2015	1.54	1.34	1.77	<0.001
YEAR = 2016	1.61	1.37	1.87	<0.001
YEAR = 2017	1.70	1.45	1.99	<0.001
YEAR = 2018	1.65	1.42	1.93	<0.001
YEAR = 2019	1.64	1.42	1.91	<0.001
YEAR = 2020	1.89	1.62	2.21	<0.001
YEAR = 2021	1.78	1.50	2.12	<0.001
YEAR = 2022	1.40	1.18	1.67	<0.001
YEAR = 2023	0.59	0.47	0.74	<0.001
MONTH = FEB	1.03	0.92	1.16	0.565
MONTH = MAR	0.93	0.84	1.03	0.169
MONTH = APR	1.07	0.96	1.20	0.202
MONTH = MAY	0.97	0.88	1.07	0.525
MONTH = JUN	0.95	0.85	1.06	0.327
MONTH = JUL	1.02	0.94	1.12	0.604
MONTH = AUG	1.00	0.90	1.10	0.914
MONTH = SEP	0.99	0.87	1.12	0.814
MONTH = OCT	0.97	0.88	1.06	0.439
MONTH = NOV	1.06	0.95	1.18	0.336
MONTH = DEC	1.04	0.93	1.15	0.507
CTSI FACULTY Prior to forming ICRE	2.04	1.45	2.88	<0.001
CTSI FACULTY Pre-Funding*	0.74	0.34	1.58	0.430
CTSI FACULTY Funded*	2.50	1.21	5.16	0.013

* Compared to ICRE Faculty prior to forming an ICRE. All else is compared to monthly publication rates in January 2010 of faculty never in an ICRE; RMINFC = Relative Monthly Increase in the Number of Future Citations; the reference category is a non-ICRE faculty in January 2010.

The modelling is completed on using quasi likelihood with log mean link and variance equal to mean (both the mean and variance link functions are the same as in Poisson distribution: log link and variance equal to mean). Sandwich variance estimator was used to account for the effect of clustering.

The sub-study reported in Supplementary Material 1, Supplementary Table 1, and Supplementary Figure 1 verified the soundness of the FCD methodology.

## Discussion

We have shown that participation in formalized team science activities, specifically team science-guided ICREs, leads to increased publication rates, even in already high-rate publishers. This increase in publication rate appears almost synchronous with the formation of the ICREs. In CTSI, pre-ensembles were eligible to apply internally for competitive grant funding, which required the team to work together to prepare a proposal, state their unmet healthcare need, and define potential approaches to address this gap. Thus, as their grants were prepared, submitted, reviewed, and awarded, these ICREs may have progressed beyond the early stages of team science collaboration, i.e., forming and norming, so that when they were funded, in general, they were immediately productive, at least by the measure of publication rate.

Publication in peer-reviewed journals is one of the most important factors determining productivity and promotion for academic faculty, however other considerations include the impact factor of the journal and order of authorship [10]. Challenging this last criterion, the number of authors per publication indexed in MEDLINE/PubMed has tripled in the past five decades, making it more difficult to be first or last senior author [11]. This may lead to faculty reluctance to participate in team science research, feeling that it would dilute their perceived contributions and negatively impact their competitiveness for promotion. The estimated 150% increase in future citations for publications supported by ICRE funding highlights the model’s potential to amplify institutional visibility and scholarly influence, which can attract competitive research funding and bolster institutional rankings [12].

In recent years, there has been a push to change promotion criteria and recognize the importance of team science and the collaborator role in scientific endeavors [11–13]. Indeed, at the Medical College of Wisconsin, a Clinician-Investigator academic pathway was recently approved to acknowledge the contributions of clinicians to traditional science activities outside the role of Principal Investigator. Specific to publications, removing the order of authorship while retaining the metric of impact factor in promotion criteria has been implemented [14]. Such an incentive may be beneficial as publications in the highest impact journals tend to have author lists averaging over 16 contributors [15].

Other measures of academic productivity could be considered to assess the benefits of formal team science activities. Quantitative measures include grant submissions and awards, rates of promotion and academic advancement, and presentations at national and international meetings [10,16]. These metrics were not used in this program evaluation due to the length of time needed to measure promotion impacts and the sample size needed to identify differences in grant applications. Presentations at meetings may be plentiful and timely, but identifying such rates in a comparison population is difficult. Moving forward, as we monitor ICRE activities, impacts in these domains may become apparent. Indeed, in the short period of time the ICRE program has been implemented, there have been 24 extramural grant submissions, totaling $54M in direct costs, with two grants already approved and funded for a total of $2.2M.

Other potential benefits of formal team science programs are more qualitative in nature and difficult to measure. These largely center around academic faculty engagement and job satisfaction. Such factors include rates of retention and measures of burnout, wellness, and flourishing [17]. Team science activities such as the described ICREs may address many of the factors noted to impact faculty decisions to remain in academic medicine. These benefits include relationships, inclusion, and trust, which are elements of collaborations reaching the performing stage. We are currently engaged in mixed methods assessments of the ICREs to identify the benefits of ICREs and the effects of intervening with groups that struggle to be productive.

Limitations in this study include the retrospective nature of the analysis leading to missing data. These included some faculty not represented in the FCD as well as potential errors in the database regarding publications. The retrospective nature also did not account for faculty who left the institution during this time span. We used the GEE Poisson model to account for variation in publication rate year-to-year, seasonally, as well as across departments and large divisions. Despite this, some additional variables may play roles in publication rates such as academic rank, years in rank, and demographics. Another potential limitation to the Poisson model is the added weight given to highly collaborative publications. For example, a publication with twelve institutional authors increments monthly publication counts for twelve faculty members, whereas a publication with two authors counts for only two. A notable limitation of this study is the exclusion of publications without available impact factors, which accounted for 19% of the dataset. This omission may affect the generalizability of the findings, particularly for disciplines where high-impact journals are less common.

The findings from this study indicate that participation in team science guided ICREs significantly enhances academic productivity, as evidenced by increased publication rates. This evidence underscores the practical benefits of formalized team science structures in academic settings. Institutions aiming to boost research output and faculty engagement should consider adopting similar models. Additionally, these results suggest that ICREs can help address the challenges of collaborative research by providing structured support and resources, thereby fostering an environment conducive to high-impact scientific contributions. Institutions aiming to implement similar team science models should prioritize adequate resource allocation, including administrative support, shared facilities, and dedicated funding for interdisciplinary initiatives, while fostering an institutional culture that rewards collaborative research.

Future research should explore the long-term effects of ICRE participation on other academic metrics, such as grant awards, promotion rates, and career satisfaction. Investigating the qualitative aspects of faculty experiences within ICREs could also provide deeper insights into the mechanisms driving increased productivity. Furthermore, expanding this research to include diverse academic institutions could help generalize the findings and refine the ICRE model for broader application [7,3,8]. Future evaluations of the ICRE model should incorporate qualitative metrics, such as faculty satisfaction, team cohesion, and retention rates, to provide a more holistic understanding of its impact beyond publication and citation metrics [18].

## Supporting information

10.1017/cts.2025.10130.sm001 Tarima et al. supplementary material Tarima et al. supplementary material

## Supplementary material

The supplementary material for this article can be found at https://doi.org/10.1017/cts.2025.10130.
