# Supplementary material for: Integrated clinical research ensembles: A pathway to increased academic productivity
Source: J Clin Transl Sci. 2025 Sep 2;9(1):e209. doi: 10.1017/cts.2025.10130 (PMC12529629; doi:10.1017/cts.2025.10130)

**Title:** Integrated Clinical Research Ensembles: A Pathway to Increased Academic Productivity

**Authors:** Sergey Tarima, John R Meurer, David Friedland, Ndidiamaka Ojiako, Michael Anello, David Zimmerman, Renee McCoy, Reza Shaker

**SUPPLEMENTARY MATERIALS**

**Supplementary material 1: Sub-study**

To evaluate how well the FCD database captures faculty publications, a sub-study was performed to compare publication capture between faculty CVs, FCD, and Flight Tracker.^1^ The Medical College of Wisconsin Institutional Review Board approved the protocol (PRO00048020). Vanderbilt Institute for Clinical and Translational Research authorized use of Flight Tracker for this research. In the summer 2023, an information letter was emailed to all Medical College of Wisconsin faculty requesting their most recent CV. Less than 3% of faculty (n = 54) shared their CVs. A chart review compared the three sources of capturing publications. Inverse probability weighting was used to extend the findings from the faculty in the sub-study to apply to the entire group of faculty. To calculate weights, we fitted a logistic regression model predicting the probability that a faculty shared his or her CV with our team. Possible predictors included annual publication frequencies as captured by FCD. We found that the model with publications frequencies from years 2012, 2013, 2019, 2021 and 2022 secured the smallest Akaike Information Criterion. The area under ROC curve was equal to ~0.77. Then, weights were defined as inverse probability of sharing their CVs. Monthly publication capture rates were calculated for CV, FCD, and Flight Tracker. Numbers of faculty publications in the sub-study were visually compared between CV and FCD using scatterplots.

The sub-study comparing CVs, FCD, and Flight Tracker was based on a convenience sample of the faculty who shared their CVs. Supplementary Table 1 shows that those who shared their CVs had higher publication rates than those who did not provide them. Across the faculty in the sub-study, there were 2,918 author-publication pairs identified by any of the three publication capture sources. CVs captured 2,484 author-publication pairs (85.1%), Flight Tracker captured 1,792 (61.4%), and FCD captured 2,842 (97.4%). The weights for inverse probability weighting were calculated using a parsimonious logistic regression to predict faculty included in the sub-study using faculty yearly publications. This logistic regression showed good predictive properties with the area under the Receiver-Operating Characteristic curve of 0.77. However, the weighted probabilities of capturing publications, 85.1%, 61.5%, and 97.3%, respectively, were almost identical to the raw rates, indicating that observed differences between the included and excluded in the sample were not related to publication capture rates. Supplementary Figure 1 shows pairwise scatterplots with a straight line determining perfect association between the number of publications captured by publication capture sources. In summary, this sub-study showed that FCD captured more faculty publications in our cohort than faculty CVs and Flight Tracker.

**Reference**

1. **Helton R, Pearson S, Hartmann K.** Flight Tracker: A REDCap tool to streamline career development grant preparation and reporting. *Journal of Clinical and Translational Science*, 2024; 8 (Suppl 1): 32. doi.org/10.1017/cts.2024.110.

**Supplementary Table 1**: **Sub-study.** Comparing annual numbers of publications between current (as of 2023) faculty included (self-selection) in the sub-study and not included.

| YEAR | Not in sub-study,  N = 1,795^1^ | In sub-study,  N = 54^1^ | p-value^2^ |
| --- | --- | --- | --- |
| 2010 | 0.00 (0.00, 1.00) | 1.00 (0.00, 3.75) | 0.005 |
| 2011 | 0.00 (0.00, 2.00) | 1.00 (0.00, 3.00) | 0.003 |
| 2012 | 0.00 (0.00, 2.00) | 2.00 (0.00, 4.00) | <0.001 |
| 2013 | 1.00 (0.00, 2.00) | 1.00 (0.00, 4.50) | 0.016 |
| 2014 | 1.00 (0.00, 2.00) | 1.00 (1.00, 4.00) | <0.001 |
| 2015 | 1.00 (0.00, 2.00) | 2.00 (0.25, 5.00) | <0.001 |
| 2016 | 1.00 (0.00, 3.00) | 3.00 (1.00, 5.75) | <0.001 |
| 2017 | 1.00 (0.00, 3.00) | 2.00 (1.00, 5.75) | <0.001 |
| 2018 | 1.00 (0.00, 3.00) | 3.00 (1.00, 5.75) | <0.001 |
| 2019 | 1.00 (0.00, 3.00) | 2.50 (1.00, 6.00) | <0.001 |
| 2020 | 1.0 (0.0, 3.0) | 4.0 (1.3, 7.0) | <0.001 |
| 2021 | 1.0 (0.0, 3.0) | 5.0 (2.0, 9.8) | <0.001 |
| 2022 | 1.0 (0.0, 3.0) | 4.0 (1.3, 9.0) | <0.001 |
| 2023 | 0.00 (0.00, 1.00) | 1.00 (0.00, 2.75) | <0.001 |

| *^1^* Median (IQR) |
| --- |
| *^2^* Wilcoxon rank sum test |

**Supplementary Figure 1 Sub-study.** Scatterplot of the Number of Publications of Individual Faculty Publications by Curriculum Vitae (Y-axis) vs Faculty Collaborative Database (X-axis). The straight line determines perfect association between numbers of publications captured by publication capture sources.


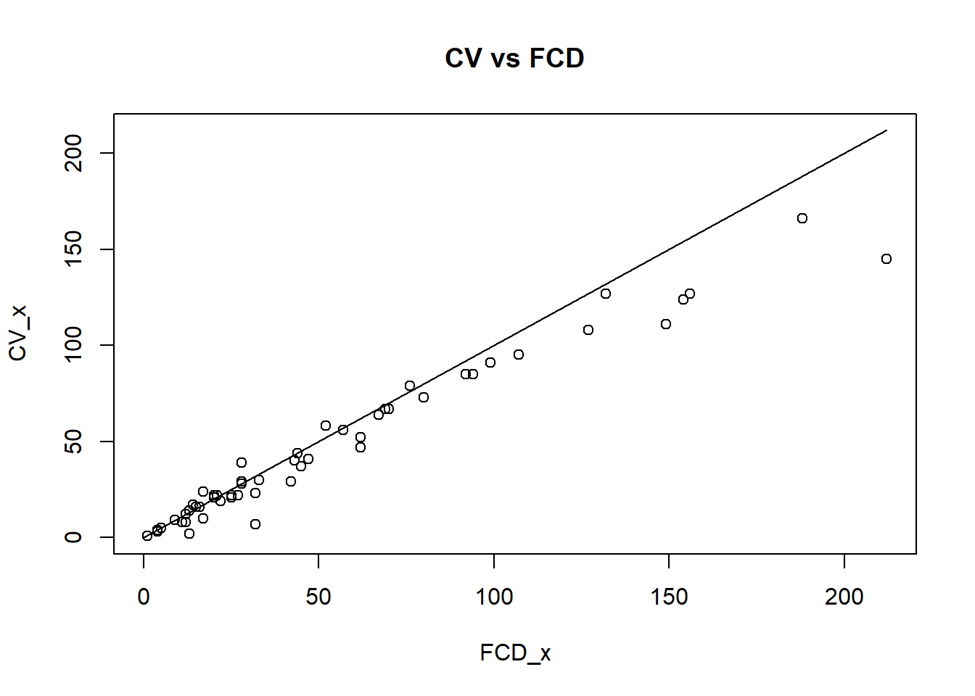

Supplement: Tarima et al. supplementary material [file S2059866125101301sup001.docx]
